# Long term effects of once-only flexible sigmoidoscopy screening after 17 years of follow-up: the UK Flexible Sigmoidoscopy Screening randomised controlled trial

**DOI:** 10.1016/S0140-6736(17)30396-3

**Published:** 2017-04-01

**Authors:** Wendy Atkin, Kate Wooldrage, D Maxwell Parkin, Ines Kralj-Hans, Eilidh MacRae, Urvi Shah, Stephen Duffy, Amanda J Cross

**Affiliations:** a Cancer Screening and Prevention Research Group, Department of Surgery and Cancer, Imperial College London, London, UK; b Clinical Trial Service Unit, The Nuffield Department of Population Health (NDPH), University of Oxford, Oxford, UK; c Centre for Cancer Prevention, Wolfson Institute of Preventive Medicine, Queen Mary University, London, UK

## Abstract

**Background:**

Colorectal cancer is the third most common cancer worldwide. Previous analyses have only reported follow-up after flexible sigmoidoscopy for a maximum of 12 years. We aimed to examine colorectal cancer incidence and mortality after a single flexible sigmoidoscopy screening and 17 years of follow-up.

**Methods:**

In this multicentre randomised trial (UK Flexible Sigmoidoscopy Screening Trial), done between Nov 14, 1994, and March 30, 1999, 170 432 eligible men and women, who had indicated on a previous questionnaire that they would probably attend screening if invited, were randomly assigned (1:2) to an intervention group (offered flexible sigmoidoscopy screening) or a control group (not contacted). Randomisation was done centrally in blocks of 12, and stratified by trial centre, general practice, and household type. The nature of the intervention did not allow the staff to be masked to arm of the trial; however, randomisation was done in batches so that the control group and participants not yet randomised were unaware of their allocation status. The primary outcomes were incidence and mortality of colorectal cancer. Hazard ratios (HRs) and 95% CIs for colorectal cancer incidence and mortality were estimated for intention-to-treat and per-protocol analyses. The trial is registered with ISRCTN, number 28352761.

**Findings:**

Our cohort analysis included 170 034 people: 112 936 in the control group and 57 098 in the intervention group, 40 621 (71%) of whom were screened and 16 477 (29%) were not screened. During screening and a median of 17·1 years' follow-up, colorectal cancer was diagnosed in 1230 individuals in the intervention group and 3253 in the control group, and 353 individuals in the intervention group versus 996 individuals in the control group died from colorectal cancer. In intention-to-treat analyses, colorectal cancer incidence was reduced by 26% (HR 0·74 [95% CI 0·70–0·80]; p<0·0001) in the intervention group versus the control group and colorectal cancer mortality was reduced by 30% (0·70 [0·62–0·79]; p<0·0001) in the intervention group versus the control group. In per-protocol analyses, adjusted for non-compliance, colorectal cancer incidence and mortality were 35% (HR 0·65 [95% CI 0·59–0·71]) and 41% (0·59 [0·49–0·70]) lower in the screened group.

**Interpretation:**

A single flexible sigmoidoscopy continues to provide substantial protection from colorectal cancer diagnosis and death, with protection lasting at least 17 years.

**Funding:**

National Institute for Health Research Efficacy and Mechanism Evaluation.

## Introduction

The UK Flexible Sigmoidoscopy Screening Trial (UKFSST) is examining the magnitude and duration of effect of a single flexible sigmoidoscopy screening, between ages 55 years and 64 years, on colorectal cancer incidence and mortality. We previously reported results from the trial, which showed that as well as being safe and well accepted, flexible sigmoidoscopy produces a substantial and sustained reduction in colorectal cancer incidence and mortality that lasts for at least 11 years.^1^, ^2^ Among those individuals attending screening in the UKFSST, colorectal cancer incidence was reduced by 33%, distal colorectal cancer incidence by 50%, and colorectal cancer mortality by 43%. Similar findings have since been reported by two further trials examining the effectiveness of a single flexible sigmoidoscopy at 55 years to 64 years, in Italy and Norway, with 11 years of follow-up.^3^, ^4^

On the basis of an independent economic analysis using the UKFSST results, the UK National Screening Committee approved the addition of a single flexible sigmoidoscopy screen at age 55 years to the English Bowel Cancer Screening Programme (BCSP) in 2011.^5^ The BCSP already included two-yearly guaiac faecal occult blood testing (gFOBT) offered from ages 60 years to 74 years. Roll out of the flexible sigmoidoscopy programme started in 2013. The aim is for full population coverage to be achieved in 2021; at the time of writing, flexible sigmoidoscopy screening was being offered by 62 (97%) of the 64 BCSP screening centres (National Health Service Bowel Cancer Screening programme, personal communication). However, flexible sigmoidoscopy screening is resource intensive and needs to have long-term efficacy in preventing colorectal cancer diagnoses and deaths to be cost-effective. Furthermore, in view of longer life expectancies, higher incidence of colorectal cancer with increasing age, and worse outcomes in older individuals,^6^ it is desirable that a single flexible sigmoidoscopy retains its efficacy into old age.

**Research in context**

**Evidence before this study**

To date, three randomised controlled trials (RCTs) have tested our hypothesis, published in *The Lancet* in 1993, that a single flexible sigmoidoscopy examination at around age 60 years is an effective strategy to prevent colorectal cancer; these are the UK Flexible Sigmoidoscopy Screening Trial (UKFSST), the Italian SCORE (Screening for COlon REctum) trial, and the Norwegian Colorectal Cancer Prevention Trial (NORCCAP). Following recruitment and screening of people aged between 55 years and 64 years, all three trials reported that the test was safe and well accepted and did not cause any increase in anxiety. In 2010, we published the major outcomes of the UKFSST study after a median follow-up of 11 years. We reported that a single flexible sigmoidoscopy examination reduced colorectal cancer incidence by 33% and colorectal cancer mortality by 43%. Subsequently, SCORE and NORCCAP published similar results, confirming the efficacy of a single flexible sigmoidoscopy after a median follow-up of 11 years. As yet, there is no published evidence on how long the protective effect of a single flexible sigmoidoscopy lasts beyond 11 years of follow-up.

**Added value of this study**

In this study we present the outcomes of the UKFSST after a median follow-up of 17 years. The UKFSST is the largest trial examining the effect of a single flexible sigmoidoscopy in reducing colorectal cancer incidence and mortality rates (n=170 432) and has a sufficient sample size to examine the effect of flexible sigmoidoscopy screening in men and women separately. Long-term follow-up is crucial to our understanding of whether the efficacy of once-only flexible sigmoidoscopy screening is sustained into older, higher colorectal cancer incidence age groups, and for determining cost-effectiveness.

Data from this large trial, where the cohort is now aged between 72 years and 81 years, shows that once-only flexible sigmoidoscopy screening continues to provide substantial protection from colorectal cancer diagnosis and death, in both men and women, with little attenuation of effect during prolonged follow-up. As a result of the continuing effectiveness of the test into older age, estimates of the number of people who need to be screened to prevent one colorectal cancer diagnosis have halved, from almost 200 (after 11 years of follow-up), to less than 100 (after 17 years of follow-up).

**Implications of all the available evidence**

The three RCTs have shown that a single flexible sigmoidoscopy is a safe, well accepted, and effective screening strategy for the prevention of colorectal cancer. Together, the trials provide important data to inform national colorectal cancer screening policy in high incidence countries. In 2010, following publication of the results of the UKFSST, the UK National Screening Committee approved the addition of a single flexible sigmoidoscopy at age 55 years to the existing NHS Bowel Cancer Screening Programme (BCSP). The BCSP already offered two yearly guaiac faecal occult blood testing between ages 60 years and 74 years.

New screening tests are always under consideration and it is essential to generate high-quality data to compare cost-effectiveness. Flexible sigmoidoscopy is an expensive and invasive test, but it has three distinct advantages over blood and stool tests. First, it is highly sensitive for small lesions and so neoplasia is detectable at a very early stage. Second, most lesions can be removed at the time of screening, so the screening in most cases is both diagnostic and therapeutic. Last, results from this study suggest that the test might not need to be repeated.

This new evidence, showing the longevity of effect of a single flexible sigmoidoscopy and the profound reduction over the past 6 years of follow-up in the number of people who need to be screened to prevent one colorectal cancer diagnosis, provides crucial data to help to inform health economic models commissioned by the UK National Screening Committee on the continued use of flexible sigmoidoscopy as part of the national BCSP in England.

We aimed to examine the efficacy of a single flexible sigmoidoscopy in reducing colorectal cancer incidence and mortality rates after a median of 17 years' follow-up overall as well as by sex and age group. We also examine the potential contaminating effect of exposure to gFOBT in the BCSP on outcomes.

## Methods

### Study design and participants

The trial rationale and methodology are described elsewhere.^1^, ^2^, ^7^, ^8^ Briefly, between Nov 14, 1994 and March 30, 1999, participants were recruited from general practices serving 14 UK hospitals. Men and women aged 55 years to 64 years were eligible to take part unless they had a history of colorectal cancer, adenomas, or inflammatory bowel disease; a life expectancy of less than 5 years; received a flexible sigmoidoscopy or colonoscopy within the previous 3 years; or were unable to provide informed consent. Eligible patients were sent a questionnaire to assess their interest in having a single flexible sigmoidoscopy screen.

Ethical approval was obtained from local research ethics committees, and all participants undergoing screening provided written informed consent.

### Randomisation and masking

Those individuals who expressed interest in having a single flexible sigmoidoscopy screen were randomly assigned to the control group or intervention group (2:1), unless they had reported meeting any of the exclusion criteria in the questionnaire, or if the required number of participants for the trial had been reached. Sequentially numbered randomisation was done centrally in blocks of 12, with stratification by trial centre, general practice, and household type. Due to the nature of the procedure masking of care staff and the intervention group was not possible. However, randomisation was done in batches so that the control group and participants not yet randomised were unaware of their allocation status. To avoid confusion within households if eligible members of the same household were allocated to different arms of the trial, potentially resulting in the wrong person attending screening, randomisation was at the household level rather than individual level.

### Procedures

The intervention group was offered flexible sigmoidoscopy screening followed by colonoscopy if high-risk polyps were found (defined as ≥1 cm; ≥3 adenomas; tubulovillous or villous histology; severe dysplasia or malignant disease; or ≥20 hyperplastic polyps above the distal rectum). The control group was not contacted.

Data for cancer registrations, date of death, cause of death, and emigrations were provided by national sources (English, Welsh and Scottish cancer registries, National Health Service [NHS] Digital, NHS National Services Scotland [NSS], the NHS Central Register [NHSCR] and the Office for National Statistics [ONS]). The cohort was matched with the NHS BCSP database and data for involvement with the programme was collected; data for invitations and completion of gFOBT kits was used for this analysis. Previous events that had not been accounted for at the time of randomisation were identified from the cancer, death, and emigration data received from national sources during follow-up, and analysis of the recent data showed changes that affected the eligibility of some participants. Where necessary, further exclusions were applied in the current analytical cohort (appendix p 1).

Colorectal cancer sites were defined by the International Classification of Diseases, 10th revision (ICD-10), and included codes C18–C20. Distal cancer was defined as C18.7, C19, and C20 (rectum and sigmoid colon) and proximal cancer as C18.0–C18.6 (descending colon to the caecum). Morphology of colorectal cancer was coded with ICD-02 codes. We included all codes relating to invasive adenocarcinomas (8140/3 8144/3, 8210/3, 8213/3, 8260/3, 8261/3, 8262/3, 8263/3, 8480/3, 8481/3, 8490/3, 8574/3), and carcinoma not otherwise specified (8000/3, 8010/3, 8020/3) for colorectal cancers that were diagnosed on clinical grounds only.

Deaths in patients with an incident colorectal cancer during follow-up were included as an endpoint in the analysis of cause-specific mortality if colorectal cancer was certified by ONS coding as the underlying cause. As certified underlying causes of deaths are still not completely accurate, with the potential for differential misattribution by trial arm,^9^ a second analysis was done after verification of assignment of colorectal cancer as the underlying cause of death for deaths that had not been certified as such by ONS. The verification was undertaken blindly by an independent expert coder (DMP) according to rules described in the appendix (p 1) to the trial results previously reported in 2010.^2^

### Outcomes

This analysis included follow-up through to Dec 31, 2014. The primary outcomes were colorectal cancer incidence and mortality, and secondary outcomes were distal colorectal cancer (rectum and sigmoid) and proximal colon cancer incidence and mortality, all-cause and non-colorectal cancer mortality, and the number needed to screen (NNS) to prevent one colorectal cancer diagnosis or death. In the estimation of incidence outcomes, only one colorectal cancer per patient was counted: the earliest colorectal cancer diagnosed for the incidence of all-site colorectal cancer and the earliest colorectal cancer in that site category for site-specific incidence. All time-to-event data were censored at emigration, death, or end of follow-up. Time to colorectal cancer and death was shown using one minus the Kaplan-Meier estimator of the survival function.

### Statistical analysis

Assuming an attendance rate at screening of 55%, two-sided significance level of α=0·05, and a Poisson distribution for the number of events, the original sample size of the trial was calculated to give 79% or more power in those aged 55–59 years and 90% or more power in those aged 60–64 years to detect a 20% difference between the intervention group and control group in colorectal cancer incidence at 10 years and mortality at 15 years since randomisation. Because screening attendance rates were greater than predicted, revised estimates indicated sufficient mortality endpoints would be acquired at 11 years.^10^

Intention-to-treat analyses, which examined the effectiveness of an invitation for screening, were undertaken using univariable Cox proportional hazards models to estimate hazard ratios (HRs) and 95% CIs. Per-protocol analyses, which examined the effectiveness of having the flexible sigmoidoscopy screening, used the method developed by Cuzick and colleagues^11^ to estimate HRs and 95% CIs adjusted for non-compliance. Annual incidence rate ratios (IRR) by years of follow-up since randomisation for all-site and distal colorectal cancer comparing intervention and control groups were estimated with exact 95% CIs for the first 16 years of follow-up, after which we had incomplete data. Per-protocol annual IRRs were estimated with adjustment for non-compliance.^11^ The NNS to prevent one colorectal cancer or one death due to colorectal cancer, with 95% CIs, were calculated using the method by Tabar and colleagues,^12^ which estimates the NNS by dividing the number attending screening by the total events prevented in the invited to screening group. Subgroup analyses were done by sex and age group (55–59 years and 60–64 years) and differences were examined using tests for interaction.

In sensitivity analyses that were not prespecified as part of the trial protocol, we examined the potential effect of contamination by gFOBT screening within the English BCSP. We identified all members of the UKFSST cohort who were screened with gFOBT as part of the BCSP and examined the effect of flexible sigmoidoscopy with and without gFOBT screening by including an indicator for gFOBT screening participation as a time varying covariate in the Cox model and performing tests of interaction. People could contribute to both not-participated and participated follow-up time, but after participation, a person was considered to remain exposed.

We also did sensitivity analyses for the outcome of distal colorectal cancer incidence where the descending colon (ICD-10 code C18.6) was included as distal. Due to the violation of the proportionality assumption for the outcomes of all-site and distal colorectal cancer incidence, we did sensitivity analyses in which we fitted exponential regression models incorporating a time threshold at 2 years, approximately where the hazard rate in the intervention group changed, and estimated the average relative rate adjusted for this. In addition, we did sensitivity analyses where the block randomisation structure was accounted for in the Cox regression models. A stratum was created for each combination of trial centre, general practice, and household type and models were fitted allowing for separate baseline hazards for each stratum as well as with standard errors adjusted for within strata correlation. Our main analyses assumed that persons within a household were independent; however, as the unit of randomisation was household and not individual, we did sensitivity analyses that accounted for the clustering by household and allowed for potentially complete dependence within households. Cox regression analyses were performed and the follow-up time of any other members of a household were censored at the date of first event within a household. No allowance for multiplicity was made in the analyses. All data were analysed with Stata 13.1.

The trial is registered with ISRCTN, number 28352761.

### Role of the funding source

The funders of the study (National Institute for Health Research Efficacy and Mechanism Evaluation) had no role in the study design, data collection, data analysis, data interpretation, or the writing of the report. Only KW, US, and WA had full access to the data. WA had final responsibility for the decision to submit for publication.

## Results

375 744 men and women aged 55–64 years were originally identified from 506 general practices of whom 7602 were excluded by their general practitioner (appendix p 1). The remaining 368 142 people were sent a questionnaire to assess their interest in having flexible sigmoidoscopy screening. Of the 194 726 (53%) expressing interest, a further 8280 were found to be ineligible and 16 014 were not required because the 40 000 participants for which the trial was funded had been reached. The remaining 170 432 were randomly assigned; 113 195 to the control group and 57 237 to the intervention group.

In both the intervention group and the control group, the mean age at randomisation was 60 years (SD 2·9) and 29 103 (51%) in the intervention group and 57 597 (51%) in the control group were women. Details of the baseline findings at screening, adverse outcomes, and satisfaction of the study participants can be found in a previous publication.^1^

Of 40 674 (71%) participants in the intervention group who underwent flexible sigmoidoscopy screening with polypectomy for small polyps, 18 were referred to surgery without colonoscopy, 2131 (5%) were referred for colonoscopy with high-risk polyps, and 38 525 (95%) were discharged with low-risk polyps or no polyps found.^1^

After the cohort was matched with the most recent data provided by national sources, 160 people were found to have died, 224 had colorectal cancer diagnosed and two had emigrated, all before the date of randomisation and were excluded from the analysis (appendix p 1). An additional person, outside of the age range, attended screening and was also excluded. Finally, 11 individuals had been randomly assigned twice (duplicate study numbers) so the second randomisation was invalidated (appendix p 1).

Our cohort analysis included 170 034 people: 112 936 in the control group and 57 098 in the intervention group, 40 621 (71%) of whom were screened and 16 477 (29%) were not screened. The median follow-up time from randomisation to death, emigration, loss to follow-up, or Dec 31, 2014, was 17·1 years (IQR 16·4–17·8; table 1).

**Table 1:** Participant characteristics by randomisation and compliance with screening

		**Control group(n=112 936)**	**Invited to screening group (n=57 098)**		
			Total (n=57 098)	Not screened (n=16 477)	Screened (n=40 621)
Age at randomisation (years)		60·0 (2·9)	60·0 (2·9)	60·1 (2·9)	60·0 (2·9)
Sex					
	Men	55 339 (49%)	27 995 (49%)	7506 (46%)	20 489 (50%)
	Women	57 597 (51%)	29 103 (51%)	8971 (54%)	20 132 (50%)
Household size					
	Single person	71 556 (63%)	36 237 (63%)	10 855 (66%)	25 382 (62%)
	Two person	41 248 (37%)	20 770 (36%)	5584 (34%)	15 186 (37%)
	Other	132 (<1%)	91 (<1%)	38 (<1%)	53 (<1%)
Length of follow-up (years)*		17·1 (16·4–17·8)	17·1 (16·4–17·8)	17·0 (15·4–17·6)	17·1 (16·6–17·9)

Data are mean (SD), n (%), or median (IQR).

* Years from date of randomisation to date of death, emigration, loss to follow-up, or Dec 31, 2014.

During screening and subsequent follow-up, 4483 people were diagnosed with colorectal cancer (3253 in the control group and 1230 in the intervention group). Distal colorectal cancers were diagnosed in 1987 people in the control group and 592 in the intervention group, of which 126 were detected at screening. Proximal colon cancers were diagnosed in 1255 people in the control group and 612 in the intervention group, with 14 detected at screening (table 2).

**Table 2:** Colorectal cancer incidence and mortality in control and intervention groups

	**Control group (n=112 936)**		**Invited to screening group (n= 57 098)**		**Hazard ratio (95% CI);intervention *vs* control group**	**p value**
	Cases	Rate (95% CI)	Cases	Rate (95% CI)		
**Incidence**						
All-site	3253*	184 (178–191)	1230*†	137 (130–145)	0·74 (0·70–0·80)	<0·0001
Distal‡	1987§	112 (107–117)	592†§	66 (61–71)	0·59 (0·54–0·64)	<0·0001
Proximal	1255§	71 (67–75)	612†§	68 (63–74)	0·96 (0·87–1·06)	0·436
**Mortality**						
Colorectal cancer¶	996‖	56 (53–60)	353‖	39 (35–43)	0·70 (0·62–0·79)	<0·0001
Distal colorectal cancer‡¶	544**	31 (28–33)	149**	17 (14–19)	0·54 (0·45–0·65)	<0·0001
Proximal colon cancer¶	403**	23 (21–25)	185**	21 (18–24)	0·91 (0·76–1·08)	0·262
Non-colorectal cancer causes¶	25 413	1427 (1410–1445)	12 926	1433 (1408–1458)	1·00 (0·98–1·03)	0·736
All cause	26 409	1483 (1465–1501)	13 279	1472 (1447–1497)	0·99 (0·97–1·01)	0·460

Rates are per 100 000 person-years.

* 108 cancers of unspecified site were included, 72 in the control group and 36 in the invited to screening group; only the earliest cancer was counted for patients with more than one cancer.

† 140 patients had cancers detected at baseline screening (126 distal cancers and 14 proximal cancers).

‡ Distal was defined as the rectum and sigmoid colon.

§ 71 patients had both a distal and a proximal cancer (30 were synchronous and 41 were metachronous): 61 patients were in the control group and 10 were in the invited to screening group.

¶ Deaths certified by the Office for National Statistics as colorectal cancer as the underlying cause of death by automatic coding.

‖ 51 deaths in patients with cancers of unspecified site were included, 36 in the control group and 15 in the invited to screening group.

** 17 deaths occurred among patients with both a proximal and a distal cancer diagnosed (13 in the control group and four in the invited to screening group) and these deaths were excluded from the site specific deaths.

**Figure 1:**
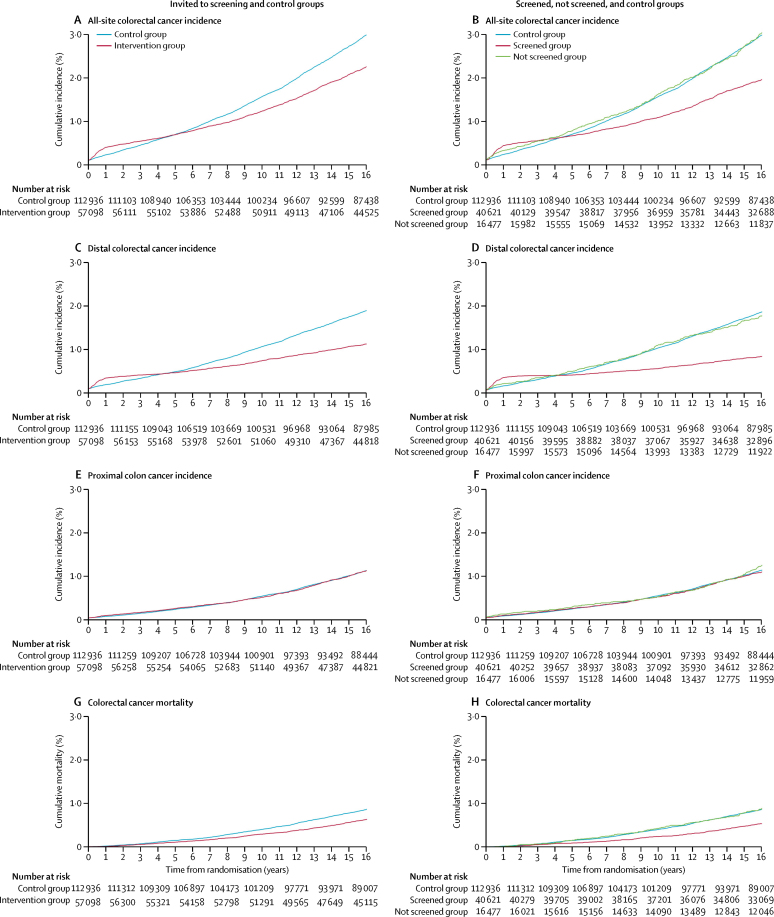
Kaplan-Meier estimates of cumulative colorectal cancer incidence and mortality

In an intention-to-treat analysis, the incidence of all-site colorectal cancer was reduced by 26% (HR 0·74 [95% CI 0·70–0·80] p<0·0001; table 2, figure 1A) and distal colorectal cancer incidence was reduced by 41% (0·59 [0·54–0·64]; p<0·0001; table 2, figure 1C) in those invited to screening. No significant effect on proximal colon cancer incidence was noted (HR 0·96 [95% CI 0·87–1·06]; p=0·436; table 2; figure 1E).

In a per-protocol analysis, which examined effectiveness in those individuals having flexible sigmoidoscopy screening, colorectal cancer incidence was reduced by 35% for all-site colorectal cancer (HR 0·65 [96% CI 0·59–0·71]) and by 56% for distal colorectal cancer (0·44 [0·38–0·50]); no significant effect on proximal colon cancer incidence was noted (0·95 [0·83–1·09]; table 3; figure 1B, 1D, 1F).

**Table 3:** Colorectal cancer incidence and mortality by randomisation and compliance with screening

	**Control group (n=112 936)**		**Invited to screening group (n=57 098)**				Hazard ratio (95% CI); screened *vs* control group*
	Cases	Rate (95% CI)	Not screened (n=16 477)		Screened (n=40 621)		
			Cases	Rate (95% CI)	Cases	Rate (95% CI)	
**Incidence**							
All-site	3253†	184 (178–191)	454†	184 (168–201)	776†‡	120 (112–128)	0·65 (0·59–0·71)
Distal§	1987¶	112 (107–117)	267¶	108 (96–121)	325‡¶	50 (45–56)	0·44 (0·38–0·50)
Proximal	1255¶	71 (67–75)	182¶	73 (63–85)	430‡¶	66 (60–73)	0·95 (0·83–1·09)
**Mortality**							
Colorectal cancer‖	996**	56 (53–59)	138**	55 (47–65)	215**	33 (29–38)	0·59 (0·49–0·70)
Distal colorectal cancer§‖	544††	31 (28–33)	83††	33 (27–41)	66††	10 (8–13)	0·34 (0·26–0·46)
Proximal colon cancer‖	403††	23 (21–25)	50††	20 (15–26)	135††	21 (17–24)	0·88 (0·70–1·10)
Non-colorectal cancer causes‖	25 413	1427 (1410–1445)	4716	1893 (1839–1948)	8210	1257 (1231–1285)	1·01 (0·97–1·04)
All cause	26 409	1483 (1465–1501)	4854	1948 (1894–2004)	8425	1290 (1263–1318)	0·99 (0·96–1·02)

Rates are per 100 000 person-years.

* Adjusted for non-compliance with screening.

† 108 cancers of unspecified site were included; 72 in the control group and 36 in the invited to screening group (eight were not screened and 28 were screened); only the earliest cancer was counted for patients with more than one cancer.

‡ 140 patients had cancers detected at baseline screening (126 distal cancers and 14 proximal cancers).

§ Distal was defined as the rectum and sigmoid colon.

¶ 71 patients had both a distal and a proximal cancer (30 were synchronous and 41 were metachronous): 61 patients in the control group and 10 patients in the invited to screening group (three were not screened and seven were screened).

‖ Deaths certified by the Office for National Statistics as colorectal cancer as the underlying cause of death by automatic coding.

** 51 deaths in patients with unspecified site cancers were included, 36 controls and 15 invited to screening (4 were not screened and 11 were screened).

†† 17 deaths occurred among patients with both a proximal and a distal cancer diagnosed (13 in the control group and four in the invited to screening group [one was not screened and three were screened]) and these deaths were excluded from the site specific deaths.

During the first 4 years after screening, cumulative incidence rates for all-site and distal colorectal cancers were higher in the intervention group than in the control group because of the detection of prevalent cancers at flexible sigmoidoscopy screening. Thereafter, the curves began to diverge as symptomatic cancers were diagnosed more frequently in the control group than in the intervention group (figure 1A, 1B, 1C, 1D). Proximal colon cancer incidence did not differ significantly between the control group and the intervention group at any time during follow-up (figure 1E, 1F).

**Figure 2:**
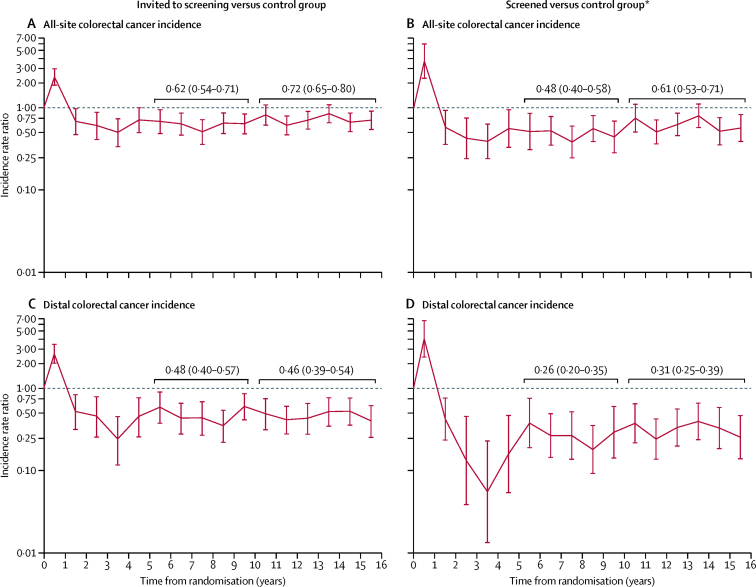
Annual incidence rate ratios (95% CI) for all-site colorectal cancer incidence and distal colorectal cancer incidence *Adjusted for non-compliance with screening.

Annual IRRs for all-site and distal colorectal cancers were below 1 throughout follow-up, except for an initial peak in the first year representing the detection of prevalent cancers at screening (figure 2). In per-protocol analyses, the average annual IRR for all-site colorectal cancer was 0·48 over years 6–10 and 0·61 over years 11–16 (figure 2B) and for distal colorectal cancer was 0·26 and 0·31, respectively (figure 2D).

The estimated NNS to prevent a single colorectal cancer diagnosis over a median of 17 years was 98 (95% CI 81–125; table 4). This compares with 191 (95% CI 145–277) after 11 years, previously published.^2^

**Table 4:** Cumulative incidence of and mortality from colorectal cancer and the number needed to screen to prevent one event in the follow-up period

		**Control group (n=112 936)**		**Invited to screening group**				**Number of events expected in intervention group**	**Number of events prevented in intervention group**	Number needed to screen to prevent one event (95% CI)*
		Cases	Rate	Total (n=57 098)		Screened (n=40 621)				
				Cases	Rate	Cases	Rate			
All participants										
	Diagnosis of colorectal cancer	3253	28·8	1230	21·5	776	19·1	1645	415	98 (81–125)
	Colorectal cancer death†	996	8·8	353	6·2	215	5·3	504	151	270 (204–397)
	Colorectal cancer death (verified) ‡	1188	10·5	416	7·3	250	6·2	601	185	220 (171–307)
Men		n=55 339		n=27 995		n=20 489				
	Diagnosis of colorectal cancer	1981	35·8	709	25·3	446	21·8	1002	293	70 (57–91)
	Colorectal cancer death†	606	11·0	206	7·4	123	6·0	307	101	204 (149–323)
	Colorectal cancer death (verified) ‡	732	13·2	244	8·7	143	7·0	370	126	162 (123–239)
Women		n=57 597		n=29 103		n=20 132				
	Diagnosis of colorectal cancer	1272	22·1	521	17·9	330	16·4	643	122	165 (113–308)
	Colorectal cancer death†	390	6·8	147	5·1	92	4·6	197	50	402 (249– 1039)
	Colorectal cancer death (verified) ‡	456	7·9	172	5·9	107	5·3	230	58	345 (220–798)
Age 55–59 years		n=56 835		n=28 561		n=20 437				
	Diagnosis of colorectal cancer	1403	24·7	521	18·2	332	16·2	705	184	111 (85–161)
	Colorectal cancer death†	422	7·4	141	4·9	77	3·8	212	71	288 (201–507)
	Colorectal cancer death (verified) ‡	501	8·8	168	5·9	94	4·6	252	84	244 (174–407)
Age 60–64 years		n=56 101		n=28 537		n=20 184				
	Diagnosis of colorectal cancer	1850	33·0	709	24·8	444	22·0	941	232	87 (68–122)
	Colorectal cancer death†	574	10·2	212	7·4	138	6·8	292	80	252 (172–470)
	Colorectal cancer death (verified) ‡	687	12·2	248	8·7	156	7·7	349	101	200 (142–330)

Rates are per 1000 persons.

* The number needed to screen was estimated using the method of Tabar and colleagues; ^11^ by dividing the number screened by the number of events prevented in the intervention group.

† Deaths certified by the Office for National Statistics as colorectal cancer as the underlying cause of death by automatic coding.

‡ Assignment of colorectal cancer as underlying cause of death by independent expert coder.

The effect of flexible sigmoidoscopy in reducing distal colorectal cancer incidence did not differ between men and women in either intention-to-treat (42% *vs* 40%, respectively) or per-protocol analysis (56% for both men and women; table 5). Flexible sigmoidoscopy had no significant effect on proximal colon cancer incidence in either men or women, whereas the reduction in all-site colorectal cancer incidence was less in women than in men in both intention-to-treat (19% *vs* 30%, p=0·0467) and per-protocol analysis (27% *vs* 40%, p=0·0480; table 5). No significant interaction was noted between flexible sigmoidoscopy and sex in effect on incidence of distal or proximal cancers, either in intention-to-treat or per-protocol analyses, but a substantial proportion of the heterogeneity by sex in the effect of flexible sigmoidoscopy on all-site colorectal cancer was due to the higher proportion of proximal cancers in women than men (45·1% *vs* 34·4%; table 5).

**Table 5:** Colorectal cancer incidence and mortality by sex, age group, randomisation, and compliance with screening

		**Control group**		**Invited to screening group**						**Hazard ratio (95% CI); invited to screening *vs* control group**	**p value**	Hazard ratio (95% CI); screened *vs* control group*	**p value**
		Cases	Rate (95% CI)	Total		Not screened		Screened					
				Cases	Rate (95% CI)	Cases	Rate (95% CI)	Cases	Rate (95% CI)				
**Incidence**													
All sites													
	Men	1981†	236 (225–246)	709†‡	166 (154–179)	263†	246 (218–277)	446†‡	140 (127–153)	0·70 (0·65–0·77)	0·0467	0·60 (0·53–0·68)	0·0480
	Women	1272†	137 (130–145)	521†‡	111 (102–121)	191†	136 (118–157)	330†‡	100 (90–112)	0·81 (0·73–0·89)		0·73 (0·63–0·84)	
	Age 55–59 years	1403†	154 (146–162)	521†‡	114 (104–124)	189†	151 (131–174)	332†‡	100 (90–111)	0·74 (0·67–0·82)	0·840	0·64 (0·56–0·74)	0·877
	Age 60–64 years	1850†	216 (207–226)	709†‡	162 (150–174)	265†	217 (193–245)	444†‡	141 (128–154)	0·75 (0·69–0·82)		0·65 (0·58–0·74)	
Distal§													
	Men	1307¶	155 (147–164)	386‡¶	90 (82–100)	170¶	158 (136–184)	216‡¶	67 (59–77)	0·58 (0·52–0·65)	0·795	0·44 (0·37–0·51)	0·981
	Women	680¶	73 (68–79)	206‡¶	44 (38–50)	97¶	69 (57–84)	109‡¶	33 (27–40)	0·60 (0·51–0·70)		0·44 (0·35–0·55)	
	Age 55–59 years	881¶	97 (90–103)	247‡¶	54 (47–61)	108¶	86 (71–104)	139‡¶	42 (35–49)	0·56 (0·48–0·64)	0·344	0·41 (0·34–0·50)	0·468
	Age 60–64 years	1106¶	129 (121–137)	345‡¶	79 (71–87)	159¶	130 (111–152)	186‡¶	59 (51–68)	0·61 (0·54–0·69)		0·46 (0·38–0·55)	
Proximal													
	Men	681¶	80 (75–87)	308‡¶	72 (64–80)	90¶	83 (68–103)	218‡¶	68 (59–78)	0·89 (0·78–1·02)	0·110	0·86 (0·71–1·03)	0·113
	Women	574¶	62 (57–67)	304‡¶	65 (58–72)	92¶	65 (53–80)	212‡¶	64 (56–74)	1·05 (0·91–1·20)		1·07 (0·87–1·31)	
	Age 55–59 years	515¶	56 (52–61)	256‡¶	56 (49–63)	77¶	61 (49–77)	179‡¶	54 (46–62)	0·99 (0·85–1·15)	0·607	0·99 (0·80–1·22)	0·619
	Age 60–64 years	740¶	86 (80–92)	356‡¶	81 (73–90)	105¶	86 (71–104)	251‡¶	79 (70–90)	0·94 (0·83–1·07)		0·92 (0·77–1·09)	
**Mortality**													
CRC‖													
	Men	606**	71 (66–77)	206**	48 (42–55)	83**	77 (62–95)	123**	38 (32–46)	0·67 (0·57–0·79)	0·417	0·55 (0·44–0·69)	0·364
	Women	390**	42 (38–46)	147**	31 (27–37)	55**	39 (30–51)	92**	28 (23–34)	0·74 (0·61–0·90)		0·65 (0·49–0·84)	
	Age 55–59 years	422**	46 (42–51)	141**	31 (26–36)	64**	51 (40–65)	77**	23 (18–29)	0·67 (0·55–0·81)	0·519	0·52 (0·39–0·69)	0·293
	Age 60–64 years	574**	66 (61–72)	212**	48 (42–55)	74**	60 (48–75)	138**	43 (37–51)	0·72 (0·62–0·84)		0·63 (0·51–0·78)	
Distal CRC§‖													
	Men	379††	45 (40–49)	98††	23 (19–28)	53††	49 (37–64)	45††	14 (10–19)	0·51 (0·41–0·64)	0·368	0·32 (0·23–0·45)	0·553
	Women	165††	18 (15–21)	51††	11 (8–14)	30††	21 (15–30)	21††	6 (4–10)	0·61 (0·45–0·83)		0·39 (0·23–0·66)	
	Age 55–59 years	242††	26 (23–30)	62††	13 (10–17)	40††	32 (23–43)	22††	7 (4–10)	0·51 (0·39–0·67)	0·560	0·30 (0·17–0·44)	0·210
	Age 60–64 years	302††	35 (31–39)	87††	20 (16–24)	43††	35 (26–47)	44††	14 (10–19)	0·56 (0·44–0·71)		0·40 (0·28–0·56)	
Proximal CC‖													
	Men	201††	24 (21–27)	97††	23 (18–28)	27††	25 (17–36)	70††	22 (17–27)	0·95 (0·75–1·21)	0·562	0·94 (0·67–1·30)	0·572
	Women	202††	22 (19–25)	88††	19 (15–23)	23††	16 (11–25)	65††	20 (15–25)	0·86 (0·67–1·10)		0·82 (0·59–1·13)	
	Age 55–59 years	158††	17 (15–20)	69††	15 (12–19)	22††	17 (11–27)	47††	14 (11–19)	0·87 (0·66–1·15)	0·735	0·82 (0·55–1·21)	0·686
	Age 60–64 years	245††	28 (25–32)	116††	26 (22–32)	28††	23 (16–33)	88††	28 (22–34)	0·93 (0·74–1·15)		0·91 (0·68–1·20)	
Non-CRC‖													
	Men	14 977	1763 (1735–1792)	7712	1793 (1753–1833)	2658	2457 (2365–2552)	5054	1569 (1527–1613)	1·02 (0·99–1·04)	0·158	1·03 (0·98–1·07)	0·168
	Women	10 436	1121 (1099–1142)	5214	1105 (1075–1135)	2058	1460 (1398–1524)	3156	954 (921–988)	0·99 (0·95–1·02)		0·98 (0·93–1·03)	
	Age 55–59 years	10 010	1092 (1071–1114)	5099	1107 (1077–1138)	1903	1511 (1444–1580)	3196	955 (923–989)	1·01 (0·98–1·05)	0·329	1·02 (0·97–1·08)	0·363
	Age 60–64 years	15 403	1783 (1755–1811)	7827	1773 (1734–1812)	2813	2283 (2200–2369)	5014	1575 (1532–1619)	0·99 (0·97–1·02)		0·99 (0·95–1·03)	
All cause													
	Men	15 583	1835 (1806–1864)	7918	1841 (1800–1881)	2741	2534 (2440–2630)	5177	1608 (1564–1652)	1·00 (0·98–1·03)	0·218	1·00 (0·96–1·05)	0·216
	Women	10 826	1163 (1141–1185)	5361	1136 (1106–1167)	2113	1499 (1436–1564)	3248	982 (948–1016)	0·98 (0·95–1·01)		0·96 (0·91–1·02)	
	Age 55–59 years	10 432	1138 (1116–1160)	5240	1138 (1107–1169)	1967	1562 (1494–1632)	3273	978 (945–1012)	1·00 (0·97–1·03)	0·423	1·00 (0·95–1·05)	0·468
	Age 60–64 years	15 977	1849 (1821–1878)	8039	1821 (1781–1861)	2887	2343 (2259–2430)	5152	1618 (1575–1663)	0·98 (0·96–1·01)		0·98 (0·94–1·02)	

Rates are per 100 000 person-years. p values are for test of interaction. Control group: men, n=55 339; women, n=57 597; age 55–59 years, n=56 835; age 60–64 years, n=56 101. Total: invited to screening group: men, n=27 995; women, n=29 103; age 55–59 years, n=28 561; age 60–64 years, n=28 537. Invited to screen group—not screened: men, n=7506; women, n=8971; age 55–59 years, n=8124; age 60–64 years, n=8353. Invited to screen group—screened: men, n=20 489; women, n=20 132; age 55–59 years, n=20 437; age 60–64 years, n=20 184. CRC=colorectal cancer. CC=colon cancer.

* Adjusted for non-compliance with screening.

† 108 site not specified cancers were included, 72 in the control group and 36 in the invited to screening group (eight were not screened and 28 were screened). Only the earliest cancer was counted for patients with more than one cancer.

‡ 140 patients had cancers detected at baseline screening (126 distal cancers and 14 proximal cancers).

§ Distal was defined as the rectum and sigmoid colon.

¶ 71 patients had both a distal and a proximal cancer (30 were synchronous and 41 were metachronous): 61 patients in the control group and 10 in the invited to screening group (three were not screened and seven were screened).

‖ Deaths certified by the Office for National Statistics as colorectal cancer as underlying cause of death by automatic coding.

** 51 deaths in patients with unspecified site cancers were included, 36 controls and 15 invited to screening (4 were not screened and 11 were screened).

†† 17 deaths occurred among patients with both a proximal and a distal cancer diagnosed (13 controls and four invited to screening [one was not screened and three were screened]) and these deaths were excluded from the site specific deaths.

The NNS to prevent a colorectal cancer diagnosis was more than twice as high for women as for men (165 [95% 113–308) *vs* 70 [57–91]). The main contributors to this were the lower incidence of colorectal cancer in the control group in women than in men (22·1 *vs* 35·8 per 1000; table 4) and the weaker effect of flexible sigmoidoscopy on all-site colorectal cancer in women than in men.

Examination of the effect of flexible sigmoidoscopy by age at randomisation showed similar findings for those aged 55–59 years and those aged 60–64 years for incidence of all colorectal cancers and by subsite in both intention-to-treat and per-protocol analyses, with no evidence for an interaction by age (table 5). For example, in intention-to-treat analyses, risk for all-site colorectal cancer was 26% lower in those offered screening aged 55–59 years compared with 25% lower in those aged 60–64 years (p=0·840); for distal cancer, the risks were 44% lower in the younger age group and 39% lower in the older age group (p=0·344). The NNS to prevent a colorectal cancer diagnosis was 111 (95% CI 85–161) in the those aged 55–59 years and 87 (68–122) in those aged 60–64 years (table 4).

The effect of age was also examined within each sex; although no significant heterogeneity was noted (appendix pp 2–3). In intention-to-treat analyses, the incidence of distal colorectal cancer was reduced by 47% in men aged 55–59 years and 37% in men aged 60–64 years; for women, the risks were reduced by 39% and 42% in each of the age groups, respectively. In per-protocol analyses, a similar pattern was noted; among men, the risk for distal colorectal cancer was 61% and 52% lower in the younger and older age groups, respectively, and among women, the risks were 53% and 59% lower, respectively.

During follow-up, 39 688 (23%) people died: 26 409 (23%) in the control group and 13 279 (23%) in the intervention group. 693 deaths were attributed to distal colorectal cancer and 588 to proximal colon cancer (544 and 149 to distal colorectal cancer and 403 and 185 to proximal colon cancer in the control and intervention groups, respectively; table 2). In an intention-to-treat analysis of certified deaths, all-site colorectal cancer mortality was reduced by 30% (HR 0·70 [95% CI 0·62–0·79]; p<0·0001); distal colorectal cancer mortality by 46% (0·54 [0·45–0·65]; p<0·0001) and proximal colon cancer mortality by a non-significant 9% (0·91 [0·76–1·08]; 0·262; table 2). An additional 255 deaths were attributed to colorectal cancer after verification by the independent coder (192 in the control group and 63 in the intervention group) but the reduction in all-site colorectal cancer mortality remained unchanged (HR 0·69 [0·62–0·77]; appendix p 4).

In the per-protocol analysis, the reduction in colorectal cancer mortality in the screened group was 41% (HR 0·59 [95% CI 0·49–0·70]; table 3). Distal colorectal cancer mortality was reduced by 66% (HR 0·34 [95% CI 0·26–0·46]), and proximal colon cancer mortality was reduced by 12%, although it did not reach statistical significance (0·88 [0·70–1·10]; table 3).

The estimated NNS to prevent one death from certified colorectal cancer over a median of 17 years was 270 (95% CI 204–397; table 4). The NNS to prevent one verified colorectal cancer death was 220 (95% CI 171–307). This compares with 489 (95% CI 343–852) and 402 (291–647), respectively, at 11 years.^2^

Interactions between flexible sigmoidoscopy and sex on all site and distal colorectal cancer mortality were not significant. All-site colorectal cancer mortality was reduced by 33% and 26% in men and women in intention-to-treat analysis, and by 45% and 35% in per-protocol analysis, whereas distal colorectal cancer mortality was reduced by 49% and 39% in intention-to-treat analysis and by 68% and 61% in per-protocol analysis, respectively (table 5). However, the NNS to prevent a colorectal cancer death was nearly twice as high for women as for men (402 [249– 1039] *vs* 204 [149–323]). This finding was due to lower colorectal cancer mortality rates in the control group in women than in the men (6·8 per 1000 *vs* 11·0 per 1000; table 4).

The effects of flexible sigmoidoscopy on colorectal cancer mortality were similar in the two categories of age at randomisation examined. For all-site colorectal cancer mortality, the risk reduction in those invited to screening was 33% in the younger age group (55–59 years) and 28% in the older age group (60–64 years); for distal cancer mortality, the risk reduction was 49% and 44% in each of the age groups, respectively (table 5). The NNS to prevent a colorectal cancer death was 288 (95% CI 201–507) in the those aged 55–59 years and 252 (172–470) in those aged 60–64 years (table 4).

The effect of age within each sex did not show any significant differences (appendix pp 2–3). In intention-to-treat analyses, the risk of distal colorectal cancer mortality was 51% lower in men aged 55–59 years and 48% lower in men aged 60–64 years; for women, the risks were 46% and 34% lower in each of the age groups, respectively. In per-protocol analyses, the risk for distal colorectal cancer mortality in men was 73% and 64% lower in the 55–59 year age group and 60–64 year age groups, respectively, and among women, the risks were 74% and 51% lower, respectively.

In a sensitivity analysis, we investigated the effect of contamination by the BCSP, by examining the effect of flexible sigmoidoscopy on colorectal cancer incidence and mortality with and without participation in gFOBT screening. About 30% of the control and intervention groups were screened using gFOBT at least once: 34 237 (30·3%) people in the control group and 16 834 (29·5%) people in the intervention group. A median of 11·3 years (IQR 10·2–12·8) had elapsed between randomisation and first screen by gFOBT, and 1577 (1·4%) people in the control group and 688 (1·2%) in the intervention group tested positive at least once. The effect of flexible sigmoidoscopy on colorectal cancer incidence and mortality did not differ significantly with and without gFOBT screening in the BCSP (appendix p 5); the reduction in all-site colorectal cancer incidence was 27% and 25% with and without participation, respectively (p_heterogeneity_=0·80), and 48% and 40%, respectively, for distal colorectal cancer (p _heterogeneity_=0·35; appendix p 5).

The effect of flexible sigmoidoscopy on distal colorectal cancer incidence and mortality was very slightly weakened when the definition of distal was expanded to also include descending colon cancers. In intention-to-treat analyses, distal colorectal cancer incidence and mortality was 39% (HR 0·61 [95% CI 0·56–0·67]) and 44% (0·56 [0·47–0·66]) lower, respectively, in the screened versus the control group (appendix p 6).

The use of models that allowed for non-proportional hazards for the analysis of the outcomes of all-site and distal colorectal cancer incidence did not provide materially different results from those generated from the Cox proportional hazards models. Additionally, the results generated from models that accounted for the block randomisation structure were similar to those from models that did not account for this. Analyses that accounted for the clustering by household provided results very similar to those presented in table 2 (appendix p 7).

## Discussion

Results of three randomised trials, including our UK study, have reported that a single flexible sigmoidoscopy examination between 55 years and 64 years of age confers a substantial reduction in colorectal cancer incidence and mortality that lasts at least 11 years.^2^, ^3^, ^4^ We now present the first randomised trial evidence that protection from a single flexible sigmoidoscopy remains substantial with even longer follow-up.

The median follow-up time in this analysis was 17 years during which incidence of colorectal cancer at all sites in screened patients was reduced by 35%, and distal cancer incidence was reduced by 56%. This compares with reductions of 33% and 50%, respectively, after a median of 11 years we reported in 2010.^2^ Consistent with this finding, the NNS to prevent one colorectal cancer diagnosis had almost halved since the last report, from 191 after 11 years of follow-up to 98 after 17 years. These results suggest that a single flexible sigmoidoscopy screen continues to provide long-lasting protection against colorectal cancer diagnosis.

The estimated reductions in incidence in the screened group include prevalent screen-detected cancers, which clearly could not be prevented by screening, and the protective effect of flexible sigmoidoscopy screening on cumulative incidence rates became apparent only after 5 years. When distal colorectal cancer incidence was analysed from five years, incidence was estimated to be reduced by 68% over the remainder of follow-up among the screened group. Evidence of the long-term efficacy of flexible sigmoidoscopy in reducing distal colorectal cancer incidence after screening is apparent from the annual IRRs which estimated an average reduction of 74% between years 6 and 10, and 69% between years 11 and 16.

Proximal colon cancer incidence and mortality was reduced by a non-significant 5% and 12%, respectively. This small effect was expected since referral for a colonoscopy following flexible sigmoidoscopy screening was restricted to those with high-risk polyps, and as a result only 5% of the screened group had a colonoscopy. Other trials had looser criteria for colonoscopy after flexible sigmoidoscopy resulting in higher rates of colonoscopy and a greater impact on proximal colon cancer.^3^, ^13^

For all-site colorectal cancers, the average incidence reduction after flexible sigmoidoscopy screening decreased from 52% over 6–10 years of follow-up, to 39% over 11–16 years. As a corresponding weakening in effect was not noted for distal colorectal cancer incidence, the attenuation seen for all-site colorectal cancer was probably due to the increasing proportion of cancers being proximally located as participants aged. Analysis of English colorectal cancer incidence statistics for 2014 showed that from age 55 years, the proportion of cancers located proximally increased from 29% at age 55–59 years to 48% at age 80–84 years.^14^ As our cohort was screened by flexible sigmoidoscopy at ages 55–64 years and the age of the participants after 17 years' follow-up ranged from 72–81 years, the proportion of proximal cancers would be expected to have increased over the years of follow-up.

We found that the reduction in distal colorectal cancer incidence after a single flexible sigmoidoscopy was the same in men and women at 56%. Since flexible sigmoidoscopy acts mainly on the distal colon this is reassuring and indicates that women are likely to benefit to the same extent as men in terms of preventing cancer of the rectum and sigmoid colon. However, we observed that the effect on all-site colorectal cancer incidence was weaker in women as have the US and the Norwegian trials of flexible sigmoidoscopy screening.^4^, ^13^ This is not surprising because rates of proximal colon cancer as a proportion of all colorectal cancers is higher in women than men particularly at older ages. For example, in England in 2014, proximal colon cancers constituted 27·5% of colon cancers in men compared with 34% in women at age 55 years, whereas at age 80 years the proportions rose to 39% and 52%, respectively.^14^

The increase in proximal cancers with age emphasises the importance of screening measures to target cancers in the proximal colon, such as colonoscopy. In a large observational study of 88 902 individuals over 22 years, colonoscopy was associated with a 27% reduction in proximal cancer incidence (multivariate HR 0·73 [95% CI 0·57–0·92).^15^ Additionally, proximal cancer incidence rates in the US decreased by 2·3–2·6% per year between 2000 and 2008, coinciding with the increased colonoscopy use since 2000 (distal colorectal cancer incidence also decreased by 2·9–3·5% per year).^16^ However, in the UK and many European countries, population screening by colonoscopy is not feasible because of competing demands on the use of colonoscopy for investigation of symptomatic patients and for surveillance of higher-risk groups. Other approved screening tests for colorectal cancer include stool occult blood tests, a stool-based multitarget DNA test, and methylated Septin9, a blood-based test.^17^, ^18^, ^19^ Although these tests detect cancers and some precursor lesions in the proximal colon,^17^, ^18^ there is very little data for the effect of these tests on proximal colon cancer incidence.

Examining our data by age at randomisation showed consistent, and significant reductions for all-site and distal colorectal cancer incidence and mortality in both the younger and older age categories when men and women were combined and within each sex. By contrast, a recent publication of a pooled analysis of three other trials reported that flexible sigmoidoscopy was not effective in women older than 60 years.^20^ In addition to the heterogeneity between studies, the findings in this recent publication were restricted to a median follow-up time between 10·5 years and 12·1 years and slightly fewer incident colorectal cancers (n=4157) than our trial (n=4483). Despite the differences in findings between the recent pooled analysis and our trial, the clinical relevance of the effect of giving a single flexible sigmoidoscopy procedure to an individual older than 60 years is restricted because it is unlikely that any screening programme would advocate the use of a single flexible sigmoidoscopy as late as aged 60 years when it is clearly effective when done at a younger age.

Incidence of colorectal cancer increases with age with 60% of colorectal cancers diagnosed after 70 years. Without cancer a person older than 70 years old is likely to live at least another decade.^6^ Older people with colorectal cancer are more likely to be diagnosed as an emergency and are less likely to be offered curative treatment than are younger people. Even if offered surgery older people are more likely to die both in the short and longer term.^6^ Our findings that an early, once-only flexible sigmoidoscopy screen reduces the chance of a colorectal cancer diagnosis in the UKFSST cohort, now aged between 72 years and 81 years, suggests that this is a promising strategy to reduce colorectal cancer and the associated health-care burden in older people.

Importantly, we have shown that flexible sigmoidoscopy is safe, with rare adverse events, which mostly occur as a result of polyp removal.^1^ Additionally, we reported in 2010 that all-cause mortality was slightly but not significantly reduced in the screened arm of the trial (HR 0·97 [95% CI 0·94–1·00]; p=0·0519), with no increase in non-colorectal cancer mortality.^2^

Our trial has several strengths. We have estimated the effect of a single flexible sigmoidoscopy screen over a long follow-up period of an average of 17 years and are the first to publish findings over such a long period. Our large sample size and the fact that 75% of the cohort was still living after a median of 17 years of follow-up since screening, provided an opportunity to look at efficacy of the test into participants' old age. We used national datasets for ascertainment of cancers, mortality, and participation in the BCSP and, as a result, have very little loss to follow-up as we could continue to follow participants even if they migrated within the UK. The BCSP provided data, which allowed us to investigate contamination with gFOBT screening and to examine the effect of participation in the programme on outcomes in the trial. In 2006, the BCSP began rollout of a programme of biennial gFOBT, with rollout complete by 2010. The programme ultimately aimed to offer screening to all men and women aged between 60 years and 74 years registered with a general practitioner. Because the BCSP roll-out was staggered by geographical area and age, a proportion of the UKFSST cohort was over age by the time they came under coverage. As a result, less than a third of the UKFSST cohort had gFOBT screening, with a similar rate of participation in the control group and the intervention group and less than 1·5% ever testing positive. Moreover, we found very little difference in the effect of flexible sigmoidoscopy on colorectal cancer incidence or mortality with and without gFOBT screening (appendix p 5).

One limitation of the trial is that the cohort was selected based on their interest in attending screening. The uptake rate was therefore higher than would be expected in a population-based programme. However, the incidence of colorectal cancer in the English participants in our control group in the first 10 years of follow-up (ie, before roll-out of the BCSP) was no different to that expected in the general population (standardised incidence ratio of 1·02 [95% CI 0·97–1·09])^14^ suggesting that our trial cohort is representative in terms of risk of colorectal cancer. Finally, improvements have been made in endoscopy standards over the past decade, so the benefit of flexible sigmoidoscopy in the future could be even greater than we estimate from our data.

We will continue to follow-up the trial cohort enabling us to monitor the long-term effects of flexible sigmoidoscopy screening on colorectal cancer incidence and mortality, and to further examine the longevity of effect. In conclusion, our results show that a single flexible sigmoidoscopy examination in people aged between 55 years and 64 years confers a substantial protection from colorectal cancer diagnosis and death lasting at least 17 years.
