# Supplementary material for: Long term effects of once-only flexible sigmoidoscopy screening after 17 years of follow-up: the UK Flexible Sigmoidoscopy Screening randomised controlled trial
Source: Lancet. 2017 Apr 1;389(10076):1299–311. doi: 10.1016/S0140-6736(17)30396-3 (PMC6168937; doi:10.1016/S0140-6736(17)30396-3)
Supplement: Supplementary appendix [file mmc1.pdf]

THE LANCET

Supplementary appendix

This appendix formed part of the original submission and has been peer reviewed. We post it as supplied by the authors.

Supplement to: Atkin W, Wooldrage K, Parkin DM, et al. Long term effects of once-only flexible sigmoidoscopy screening after 17 years of follow-up: the UK Flexible Sigmoidoscopy Screening randomised controlled trial. *Lancet* 2017; published online Feb 21. http://dx.doi.org/10.1016/S0140-6736(17)30396-3.

Supplementary Figure 1: Trial Profile

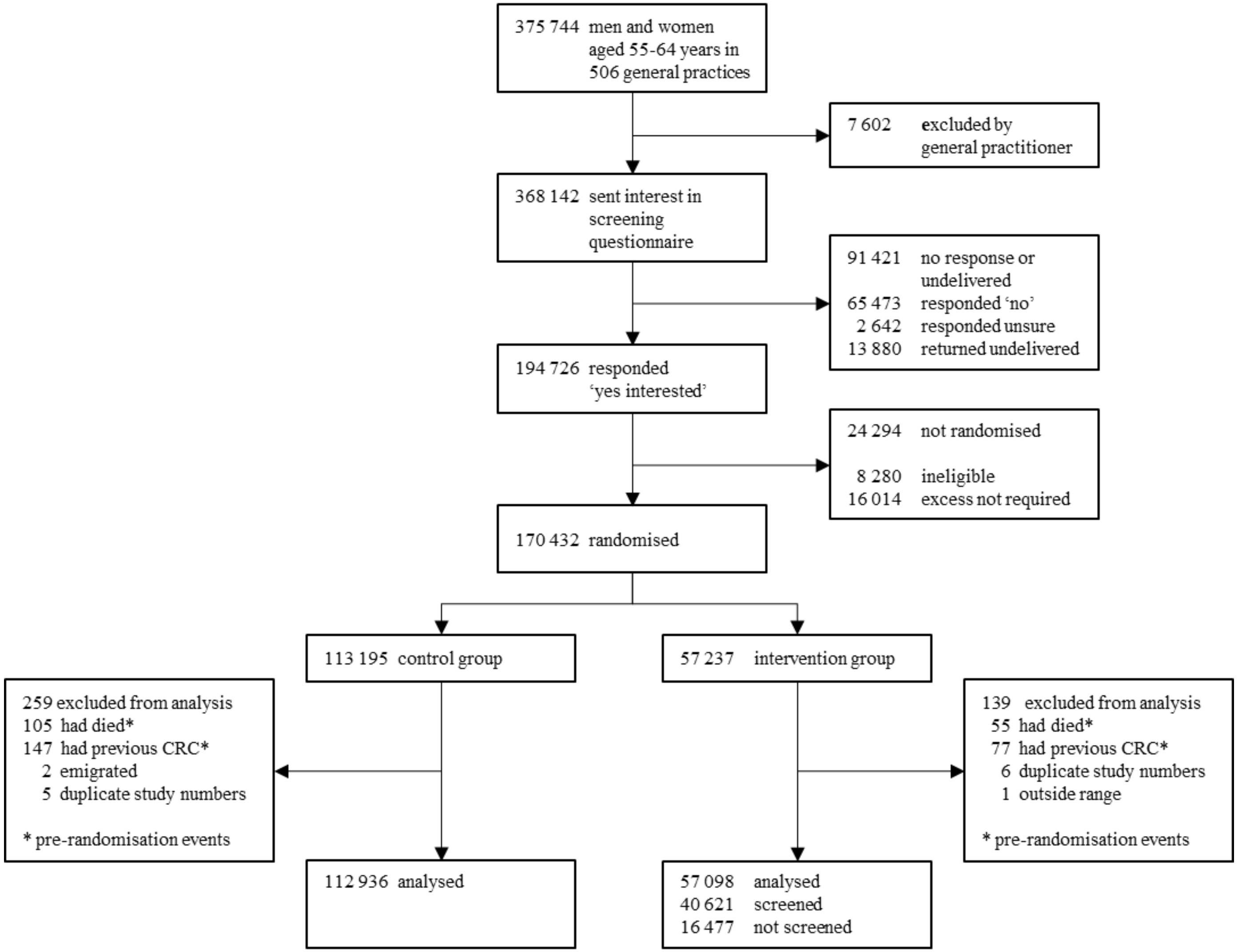

Supplementary Table 1. Colorectal cancer incidence and mortality by sex and age groups, randomisation and compliance with screening

		Control group (Men: 55-59yrs n=27,706, 60-64yrs n=27,633 Women: 55-59yrs n=29,129, 60-64yrs n=28,468)		Invited to screening group: Total (Men: 55-59yrs n=13,991, 60-64yrs n=14,004 Women: 55-59yrs n=14,570, 60-64yrs n=14,533)		Invited to screening group: Not screened (Men: 55-59yrs n=3,691, 60-64yrs n=3,815 Women: 55-59yrs n=4,433, 60-64yrs n=4,538)		Invited to screening group: Screened (Men: 55-59yrs n=10,300, 60-64yrs n=10,189 Women: 55-59yrs n=10,137, 60-64yrs n=9,995)		Hazard ratio (95% CI); invited to screening vs control group	p-value	Hazard ratio (95% CI); screened vs control group*	p-value
		**Cases**	**Rate (95% CI)**	**Cases**	**Rate (95% CI)**	**Cases**	**Rate (95% CI)**	**Cases**	**Rate (95% CI)**				
**Incidence**													
All sites													
Men	55-59yrs	858†	198 (185-212)	297†‡	135 (121-151)	108†	197 (163-238)	189†‡	115 (99-132)	0.68 (0.60-0.78)	0.530	0.58 (0.48-0.69)	0.578
	60-64yrs	1,123†	276 (260-292)	412†‡	199 (181-219)	155†	297 (253-347)	257†‡	166 (147-188)	0.72 (0.64-0.81)		0.62 (0.53-0.72)	
Women	55-59yrs	545†	114 (105-124)	224†‡	94 (82-107)	81†	115 (93-143)	143†‡	85 (72-100)	0.82 (0.70-0.96)	0.717	0.75 (0.60-0.93)	0.711
	60-64yrs	727†	162 (151-174)	297†‡	129 (115-144)	110†	158 (131-190)	187†‡	116 (100-134)	0.79 (0.69-0.91)		0.71 (0.58-0.86)	
Distal§													
Men	55-59yrs	590¶	136 (125-147)	158‡¶	72 (61-84)	69¶	126 (99-159)	89‡¶	54 (44-66)	0.53 (0.44-0.63)	0.146	0.39 (0.30-0.49)	0.180
	60-64yrs	717¶	175 (163-189)	228‡¶	110 (96-125)	101¶	193 (158-234)	127‡¶	82 (69-97)	0.63 (0.54-0.73)		0.48 (0.39-0.60)	
Women	55-59yrs	291¶	61 (54-68)	89‡¶	37 (30-46)	39¶	55 (40-76)	50‡¶	30 (23-39)	0.61 (0.48-0.78)	0.763	0.47 (0.33-0.66)	0.591
	60-64yrs	389¶	87 (78-96)	117‡¶	50 (42-61)	58¶	83 (64-107)	59‡¶	36 (28-47)	0.58 (0.47-0.72)		0.41 (0.30-0.56)	
Proximal													
Men	55-59yrs	264¶	61 (54-68)	127‡¶	58 (48-69)	35¶	64 (46-89)	92‡¶	56 (45-68)	0.95 (0.77-1.18)	0.445	0.93 (0.70-1.24)	0.432
	60-64yrs	417¶	102 (92-112)	181‡¶	87 (75-101)	55¶	104 (80-136)	126‡¶	81 (68-96)	0.85 (0.72-1.02)		0.80 (0.64-1.02)	
Women	55-59yrs	251¶	52 (46-59)	129‡¶	54 (45-64)	42¶	60 (44-81)	87‡¶	52 (42-64)	1.03 (0.83-1.27)	0.884	1.04 (0.76-1.44)	0.884
	60-64yrs	323¶	72 (64-80)	175‡¶	76 (65-88)	50¶	71 (54-94)	125‡¶	77 (65-92)	1.05 (0.87-1.26)		1.08 (0.83-1.40)	
**Mortality**													
CRC**													
Men	55-59yrs	258††	59 (52-67)	84††	38 (31-47)	38††	69 (50-95)	46††	28 (21-37)	0.64 (0.50-0.82)	0.666	0.50 (0.35-0.71)	0.485
	60-64yrs	348††	84 (76-94)	122††	58 (49-70)	45††	85 (63-114)	77††	49 (39-62)	0.69 (0.56-0.85)		0.59 (0.44-0.78)	
Women	55-59yrs	164††	34 (29-40)	57††	24 (18-31)	26††	37 (25-54)	31††	18 (13-26)	0.70 (0.52-0.94)	0.601	0.55 (0.35-0.87)	0.419
	60-64yrs	226††	50 (44-57)	90††	39 (31-48)	29††	41 (29-59)	61††	38 (29-48)	0.77 (0.60-0.99)		0.70 (0.50-0.97)	
Distal CRC§**													
Men	55-59yrs	168‡‡	38 (33-45)	42‡‡	19 (14-26)	26‡‡	47 (32-69)	16‡‡	10 (6-16)	0.49 (0.35-0.69)	0.804	0.27 (0.15-0.48)	0.421
	60-64yrs	211‡‡	51 (45-59)	56‡‡	27 (21-35)	27‡‡	51 (35-74)	29‡‡	19 (13-27)	0.52 (0.39-0.70)		0.36 (0.24-0.55)	

Women	55-59yrs	74‡‡	15 (12-19)	20‡‡	8 (5-13)	14‡‡	20 (12-33)	6‡‡	4 (2-8)	0.54 (0.33-0.89)	0.539	0.26 (0.10-0.67)	0.284
	60-64yrs	91‡‡	20 (16-25)	31‡‡	13 (9-19)	16‡‡	23 (14-37)	15‡‡	9 (6-15)	0.66 (0.44-0.99)		0.49 (0.25-0.93)	
Proximal CC**													
Men	55-59yrs	78‡‡	18 (14-22)	36‡‡	16 (12-23)	10‡‡	18 (10-34)	26‡‡	16 (11-23)	0.91 (0.61-1.35)	0.789	0.88 (0.52-1.50)	0.786
	60-64yrs	123‡‡	30 (25-36)	61‡‡	29 (23-38)	17‡‡	32 (20-52)	44‡‡	28 (21-38)	0.98 (0.72-1.33)		0.97 (0.64-1.48)	
Women	55-59yrs	80‡‡	17 (13-21)	33‡‡	14 (10-19)	12‡‡	17 (10-30)	21‡‡	12 (8-19)	0.83 (0.55-1.24)	0.832	0.75 (0.42-1.35)	0.731
	60-64yrs	122‡‡	27 (23-32)	55‡‡	24 (18-31)	11‡‡	16 (9-28)	44‡‡	27 (20-36)	0.87 (0.64-1.20)		0.85 (0.58-1.25)	
Non-CRC**													
Men	55-59yrs	5,939	1358 (1324-1393)	3,027	1368 (1321-1418)	1,077	1950 (1837-2070)	1,950	1175 (1124-1228)	1.01 (0.96-1.05)	0.628	1.01 (0.95-1.08)	0.634
	60-64yrs	9,038	2193 (2149-2239)	4,685	2241 (2178-2307)	1,581	2985 (2842-3136)	3,104	1989 (1920-2060)	1.02 (0.99-1.06)		1.03 (0.98-1.09)	
Women	55-59yrs	4,071	849 (824-876)	2,072	866 (829-904)	826	1168 (1091-1250)	1,246	739 (699-781)	1.02 (0.97-1.08)	0.063	1.03 (0.94-1.13)	0.790
	60-64yrs	6,365	1409 (1374-1444)	3,142	1351 (1305-1399)	1,232	1754 (1659-1855)	1,910	1177 (1125-1231)	0.96 (0.92-1.00)		0.93 (0.87-1.00)	
All cause													
Men	55-59yrs	6,197	1417 (1382-1453)	3,111	1406 (1358-1457)	1,115	2019 (1904-2141)	1,996	1203 (1151-1257)	0.99 (0.95-1.04)	0.547	0.99 (0.92-1.06)	0.545
	60-64yrs	9,386	2278 (2232-2324)	4,807	2300 (2236-2366)	1,626	3070 (2925-3223)	3,181	2038 (1969-2110)	1.01 (0.98-1.05)		1.01 (0.96-1.07)	
Women	55-59yrs	4,235	883 (857-911)	2,129	890 (853-928)	852	1204 (1126-1288)	1,277	757 (717-800)	1.01 (0.96-1.06)	0.086	1.01 (0.93-1.10)	0.107
	60-64yrs	6,591	1459 (1424-1494)	3,232	1390 (1343-1439)	1,261	1795 (1699-1897)	1,971	1215 (1162-1269)	0.95 (0.91-0.99)		0.93 (0.87-0.99)	

Rates are per 100,000 person-years.
P-values are for test of interaction.
CRC=colorectal cancer. CC=colon cancer.

*Adjusted for non-compliance with screening. †108 site not specified cancers were included, 72 in controls and 36 in the invited to screening group (8 were not screened and 28 were screened). Only the earliest cancer was counted for patients with more than one cancer. ‡140 patients had cancers detected at baseline screening (126 distal cancers and 14 proximal cancers). §Distal was defined as the rectum and sigmoid colon. ¶71 patients had both a distal and a proximal cancer (30 were synchronous and 41 were metachronous): 61 patients were controls and 10 were in the invited to screening group (3 were not screened and 7 were screened). **Deaths certified by the Office for National Statistics as colorectal cancer as underlying cause of death by automatic coding. ††51 deaths in patients with unspecified site cancers were included, 36 controls and 15 invited to screening (4 were not screened and 11 were screened). ‡‡17 deaths occurred among patients with both a proximal and a distal cancer diagnosed (13 controls and 4 invited to screening [1 was not screened and 3 were screened]) and these deaths were excluded from the site specific deaths.

Supplementary Table 2. Verified colorectal cancer mortality in control and intervention groups and by gender

	Control group (n=112,936)		Invited to screening group: Total (n=57,098)		Invited to screening group: Not screened (n=16,477)		Invited to screening group: Screened (n=40,621)		Hazard ratio (95% CI); invited to screening vs control group	p-value	Hazard ratio (95% CI); screened vs control group*	p-value
	Cases	Rate (95% CI)	Cases	Rate (95% CI)	Cases	Rate (95% CI)	Cases	Rate (95% CI)				
**CRC mortality†**												
All participants	1188	67 (63-71)	416	46 (42-51)	166	67 (57-78)	250	38 (34-43)	0.69 (0.62-0.77)	<0.0001	0.57 (0.49-0.67)	<0.0001
Men	732	86 (80-93)	244	57 (50-64)	101	93 (77-113)	143	44 (38-52)	0.66 (0.57-0.76)	0.291‡	0.53 (0.43-0.65)	0.234‡
Women	456	49 (45-54)	172	36 (31-42)	65	46 (36-59)	107	32 (27-39)	0.74 (0.62-0.89)		0.64 (0.50-0.83)	
Age 55-59yrs	501	55 (50-60)	168	36 (31-42)	74	59 (47-74)	94	28 (23-34)	0.67 (0.56-0.80)	0.637‡	0.53 (0.41-0.68)	0.435‡
Age 60-64yrs	687	80 (74-86)	248	56 (50-64)	92	75 (61-92)	156	49 (42-57)	0.71 (0.61-0.82)		0.60 (0.49-0.74)	
**Non-CRC mortality†**												
All participants	25221	1416 (1399-1434)	12863	1426 (1402-1451)	4688	1881 (1828-1936)	8175	1252 (1225-1280)	1.01 (0.99-1.03)	0.558	1.01 (0.98-1.05)	0.538
Men	14851	1748 (1721-1777)	7674	1784 (1744-1824)	2640	2440 (2349-2535)	5034	1563 (1521-1607)	1.02 (0.99-1.05)	0.134‡	1.03 (0.99-1.08)	0.146‡
Women	10370	1114 (1092-1135)	5189	1100 (1070-1130)	2048	1453 (1391-1517)	3141	949 (917-983)	0.99 (0.95-1.02)		0.98 (0.93-1.03)	
Age 55-59yrs	9931	1083 (1062-1105)	5,072	1101 (1071-1132)	1893	1503 (1437-1572)	3179	950 (918-984)	1.02 (0.98-1.05)	0.333‡	1.03 (0.97-1.08)	0.366‡
Age 60-64yrs	15290	1770 (1742-1798)	7,791	1764 (1726-1804)	2795	2269 (2186-2354)	4996	1569 (1526-1614)	1.00 (0.97-1.02)		1.00 (0.95-1.04)	

Rates are per 100,000 person-years.
CRC=colorectal cancer.

*Adjusted for non-compliance with screening. † Assignment of colorectal cancer as underlying cause of death by independent expert coder. ‡ P-value for test of heterogeneity of effect by gender or by age group.

Supplementary Table 3. Colorectal cancer incidence and mortality in control and intervention groups by participation in guaiac faecal occult blood screening (gFOBT) within the Bowel Cancer Screening Programme (BCSP)

	Control group (n = 112,936)			Invited to screening group (n = 57,098)			Hazard ratio (95% CI); invited to screening vs control group	p-value
	Cases	Person-years	Rate (95% CI)	Cases	Person-years	Rate (95% CI)		
**By participation in gFOBT screening within the BCSP***								
All-site colorectal cancer incidence								
Not participated	2,842	1,576,329	180 (174-187)	1,084	803,146	135 (127-143)	0.75 (0.70 – 0.80)	0.798
Participated	411	189,766	217 (197-239)	146	92,720	157 (134-185)	0.73 (0.60 – 0.88)	
Distal colorectal cancer incidence								
Not participated	1,757	1,580,613	111 (106-116)	534	805,569	66 (61-72)	0.60 (0.54 – 0.66)	0.353
Participated	230	190,171	121 (106-138)	58	92,924	62 (48-81)	0.52 (0.39 – 0.69)	
Proximal colon cancer incidence								
Not participated	1,078	1,585,450	68 (64-72)	527	806,658	65 (60-71)	0.96 (0.86 – 1.06)	0.847
Participated	177	190,457	93 (80-108)	85	92,900	91 (74-113)	0.98 (0.76 – 1.27)	
Colorectal cancer mortality†								
Not participated	917	1,589,869	58 (54-62)	323	809,093	40 (36-45)	0.69 (0.61 – 0.78)	0.589
Participated	79	190,869	41 (33-52)	30	93,105	32 (23-46)	0.78 (0.51 – 1.19)	
Distal colorectal cancer mortality†								
Not participated	511	1,589,869	32 (29-35)	143	809,093	18 (15-21)	0.55 (0.46 - 0.66)	0.378
Participated	33	190,869	17 (12-24)	6	93,105	6 (3-14)	0.37 (0.16 - 0.89)	
Proximal colorectal cancer mortality†								
Not participated	364	1,589,869	23 (21-25)	164	809,093	20 (17-24)	0.88 (0.73 – 1.06)	0.437
Participated	39	190,869	20 (15-28)	21	93,105	23 (15-35)	1.10 (0.65 - 1.88)	

Rates are per 100,000 person-years.
P-values are for test for heterogeneity of effect by participation in the BCSP.

*Of the control group, 78,699 (69.7%) did not participate and 34,237 (30.3%) did participate. Of the invited to screening group, 40,264 (70.5%) did not participate and 16,834 (29.5%) did participate. Participation was counted from the time a participant returned their first kit. People could contribute person-years to both not participated and participated follow-up time but after participation a person was considered to remain exposed. † Deaths certified by the Office for National Statistics as colorectal cancer as underlying cause of death by automatic coding
.

Supplementary Table 4. Distal colorectal and proximal colon cancer incidence and mortality by randomisation and compliance with screening where distal colorectal also includes the descending colon

	Control group (n=112,936)		Invited to screening group: Total (n=57,098)		Invited to screening group: Not screened (n=16,477)		Invited to screening group: Screened (n=40,621)		Hazard ratio (95% CI); invited to screening vs control group	p-value	Hazard ratio (95% CI); screened vs control group*	p-value
	Cases	Rate (95% CI)	Cases	Rate (95% CI)	Cases	Rate (95% CI)	Cases	Rate (95% CI)				
Incidence												
Distal†	2,080‡	117 (113-123)	647‡§	72 (67-78)	285‡	115 (102-129)	362‡§	56 (50-62)	0.61 (0.56-0.67)	<0.0001	0.47 (0.41-0.53)	<0.0001
Proximal	1,166‡	66 (62-70)	556‡§	62 (57-67)	163‡	66 (56-76)	393‡§	60 (55-67)	0.94 (0.85-1.04)	0.235	0.92 (0.80-1.06)	0.235
Mortality												
Distal CRC†¶	573**	32 (30-35)	162**	18 (15-21)	88**	35 (29-44)	74**	11 (9-14)	0.56 (0.47-0.66)	<0.0001	0.37 (0.28-0.48)	<0.0001
Proximal CC¶	372**	21 (19-23)	172**	19 (16-22)	45**	18 (13-24)	127**	19 (16-23)	0.91 (0.76-1.09)	0.316	0.89 (0.70-1.12)	0.315

Rates are per 100,000 person-years.
CRC=colorectal cancer.
CC=colon cancer.

* Adjusted for non-compliance with screening. †Distal was defined as the rectum, sigmoid and descending colon. ‡74 patients had both a distal and a proximal cancer (29 were synchronous and 45 were metachronous): 65 patients were controls and 9 were in the invited to screening group (2 were not screened and 7 were screened). §140 patients had cancers detected at baseline screening (133 distal cancers and 7 proximal cancers). ¶Deaths certified by the Office for National Statistics as colorectal cancer as underlying cause of death by automatic coding. **19 deaths occurred among patients with both a proximal and a distal cancer diagnosed (15 controls and 4 invited to screening [1 was not screened and 3 were screened]) and these deaths were excluded from the site specific deaths.

Supplementary Table 5. Comparison of colorectal cancer incidence and mortality in control and intervention groups analysed with censoring at first event in household

	Hazard ratio (95% CI); intervention vs control group*	p-value
**Incidence**		
All-site	0.75 (0.70 - 0.80)	<0.0001
Distal‡	0.59 (0.54 - 0.64)	<0.0001
Proximal	0.96 (0.87 - 1.06)	0.434
**Mortality**		
Colorectal cancer¶	0.70 (0.62 - 0.79)	<0.0001
Distal colorectal cancer‡¶	0.54 (0.45 - 0.65)	<0.0001
Proximal colon cancer¶	0.91 (0.76 - 1.08)	0.261
Non-colorectal cancer causes¶	1.00 (0.98 - 1.02)	0.858
All cause	0.99 (0.97 - 1.01)	0.391

*Hazard ratios estimated from Cox proportional hazards models where, for each outcome, the follow-up time of other members of a household were censored at the date of first outcome in the household.
‡Distal was defined as the rectum and sigmoid colon. ¶Deaths certified by the Office for National Statistics as colorectal cancer as underlying cause of death by automatic coding.
